# Myasthenia Gravis Complicated by M-proteinemia and Demyelinating Neuropathy: A Report of Two Cases

**DOI:** 10.7759/cureus.74886

**Published:** 2024-12-01

**Authors:** Kaiki Kawakita, Takuya Saito, Yoshiyuki Kondo, Tsuyoshi Uchiyama, Keishiro Sato

**Affiliations:** 1 Department of Neurology, Seirei Hamamatsu General Hospital, Hamamatsu, JPN

**Keywords:** immunosuppressive agents, m-proteinemia, myasthenia gravis, neuropathy, thymectomy

## Abstract

Myasthenia gravis (MG) is characterized by weakness and rapid fatigue of voluntary muscles. Here, we present two cases of early-onset MG, complicated with M-proteinemia and demyelinating neuropathy. Case one was diagnosed with MG at age 29, received tacrolimus post-thymectomy, and developed M-proteinemia and demyelinating neuropathies at age 66 before being diagnosed with B-cell lymphoma. Case two was diagnosed with MG at age 21, received immunosuppressive drugs post-thymectomy, and was diagnosed with myelin-associated glycoprotein antibody-associated neuropathy at age 66. Demyelinating neuropathy with M-proteinuria should be considered when motor symptoms worsen in patients with early-onset MG who receive immunosuppressive drugs after thymectomy.

## Introduction

Myasthenia gravis (MG) is an autoimmune disease in which antibodies that inhibit the neuromuscular junction lead to muscle weakness, fatigue, ptosis, and diplopia. Patients aged < 50 years who develop MG are classified as having early-onset MG. Most early-onset MG cases are positive for anti-acetylcholine receptor antibodies, and some patients develop thymic hyperplasia [1]. Steroids, pyridostigmine, immunosuppressive drugs, and thymectomies are the commonly administered therapies for early-onset MG [2]. Musculoskeletal symptoms and gait disorders in patients with MG are considered to be an exacerbation of MG. Here, we report two cases of early-onset MG complicated by M-proteinemia and neuropathy with late-onset musculoskeletal symptoms and gait disturbances.

## Case presentation

Case one

**Figure 1:**
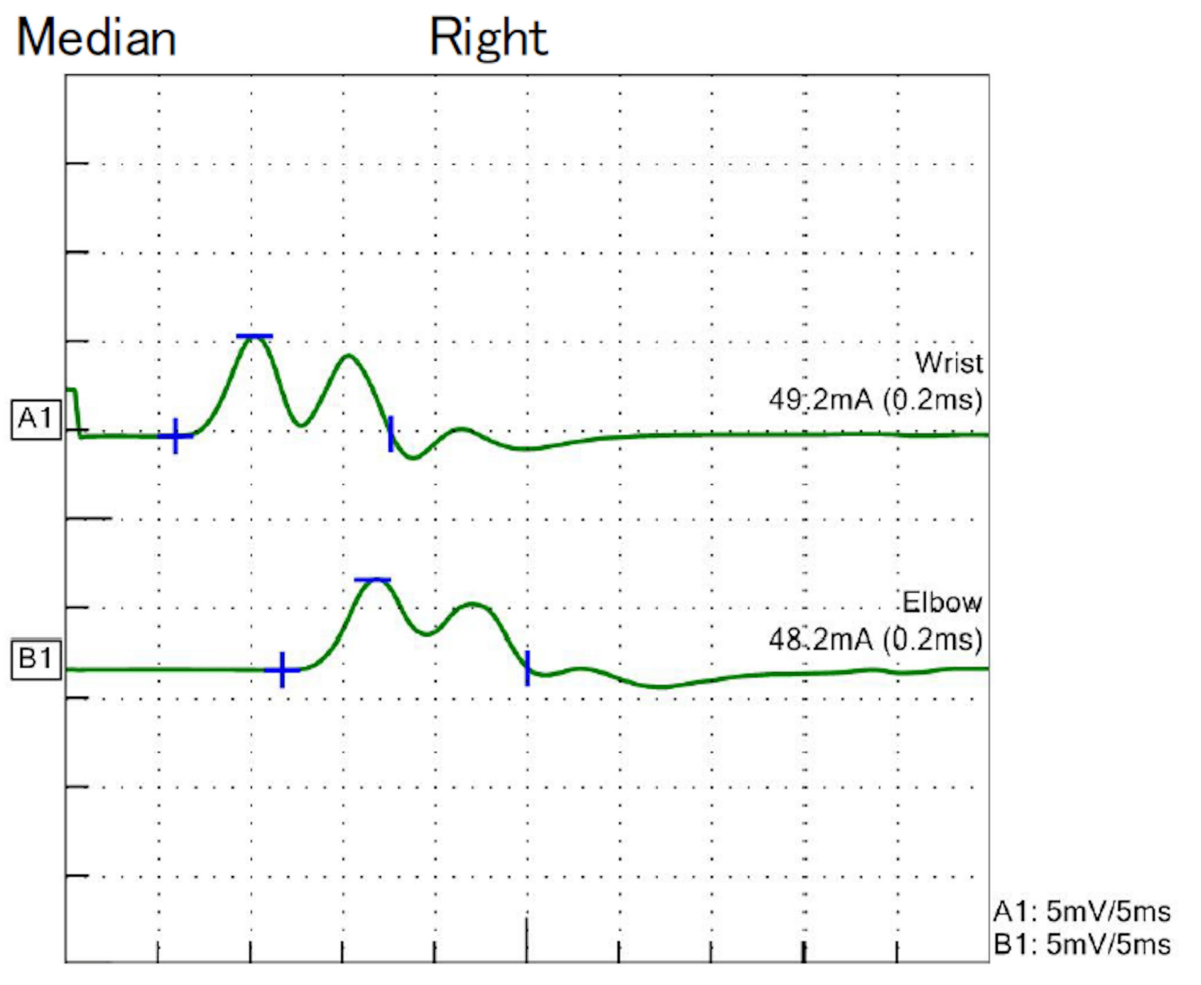
Waveform depicted by the right median nerve in NCS for case one NCS: Nerve conduction studies

The patient was a 66-year-old woman diagnosed with early-onset MG at the age of 29 years with ptosis and weakness of the limb muscles. Prednisolone (PSL) and tacrolimus (TAC) were administered, and thymectomy was performed at age 33 years. The PSL dose was tapered off without symptom worsening, and the oral TAC therapy was continued. At the age of 61 years, she had no strength in her toes and could not walk adequately. Physical examination revealed generalized mild muscle weakness in the neck and proximal and distal limbs. The deep tendon reflexes were decreased, and the sensory findings were unremarkable. Blood test results revealed the presence of M-proteinemia of IgG kappa type, and CSF analysis revealed elevated protein levels (Table 1). Nerve conduction studies (NCS) revealed demyelination of multiple nerves (Table 2, Figure 1).

**Table 1:** Results of the blood and CSF analysis in case one Hb: Hemoglobin, HbA1c: Glycated hemoglobin

Blood examination	Results	Normal range
White blood cells (/µL)	3870	3300-8600
Hb (g/dL)	11.9	11.6-14.8
Platelets (×10^4^ /µL)	29.8	15.8-34.8
Creatinine (mg/dL)	0.44	0.46-0.79
HbA_1c_ (%)	5.5	4.3-5.8
C-reactive protein (mg/dL)	0.19	0.00-0.14
Soluble interleukin-2 receptor (U/mL)	926	157-474
Antinuclear antibody (fold)	<40	<40
Anti-double-stranded DNA IgG antibody (U/mL)	<10	<12
Anti-Sjögren's syndrome-A antibody (U/mL)	≦1	<10
Anti-Sjögren's syndrome-B antibody (U/mL)	≦1	<10
Cytoplasmic anti-neutrophil cytoplasmic antibody (IU/mL)	≦1	<3.5
Perinuclear anti-neutrophil cytoplasmic antibody (IU/mL)	≦1	<3.5
Anti-acetylcholine receptor antibody (nmol/L)	0.8	<0.2
CSF analysis	Results	Normal range
Protein (mg/dL)	274	15-45
Glucose (mg/dL)	71	50-75
White blood cell count (/µL)	7	<5
Mononuclear cell (/µL)	6	
Polymorphonuclear cell (/µL)	1	
Albumin (mg/dL)	138	4.1-5.1
IgG (mg/dL)	63.7	
IgG index	0.565	<0.7

**Table 2:** Results of NCS in case one NCS: Nerve conduction studies

Nerve	Distal latency	Amplitude distal/proximal	Velocity	F-latency
Motor	ms	mV	m/s	ms
Median	6.0	5.7 / 5.1	31.9	39.1
Ulnar	5.4	3.6 / 2.9	27.1	34.3
Tibial	7.9	2.5 / 2.0	24.1	Not evoked
Sensory	ms	μV	m/s	ms
Median	6.1	3.0 / 2.7	22.9	Not evoked
Sural	5.0	6.8	25.9	Not evoked

The results of repeated stimulation tests were negative. Chronic inflammatory demyelinating polyneuropathy with monoclonal gammopathy of undetermined significance was diagnosed, and intravenous immunoglobulin (IVIG) was administered. Her symptoms partially improved after the IVIG therapy. At the age of 63 years, she developed lymphadenopathy in her neck and both axillae. Subsequently, the patient was diagnosed with diffuse large B-cell lymphoma (DLBCL).

Case two

**Figure 2:**
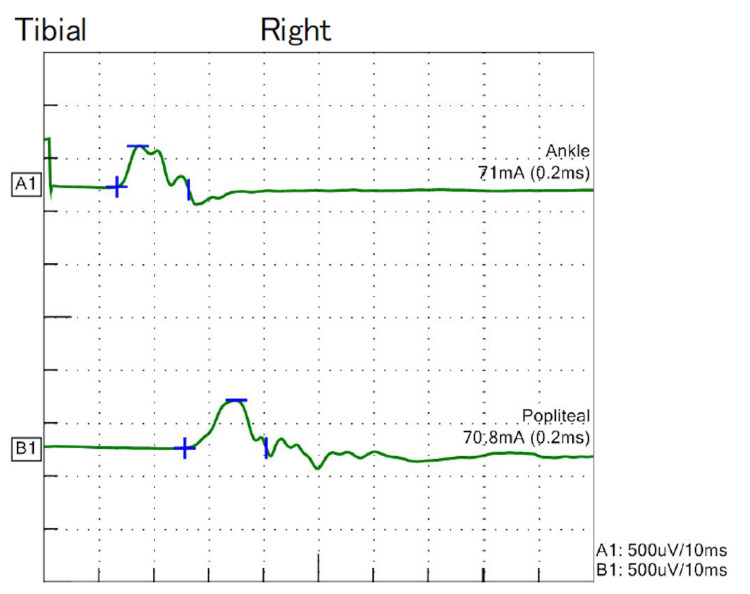
Waveform depicted by the right tibial nerve in NCS for case 2 NCS: Nerve conduction studies

The patient involved was a 67-year-old woman who was diagnosed with early-onset MG at the age of 21 years with diplopia and limb weakness. Prednisolone and azathioprine (AZA) were administered, and a thymectomy was performed at 22 years of age. Plasma exchange was repeated because of the exacerbation of MG symptoms. Immunosuppressive drugs caused side effects, and the medication was changed from AZA to cyclosporine and then to TAC. However, administration of PSL 10 mg and TAC was continued for a long period. At the age of 67 years, she complained of discomfort in her feet and hands, as if walking on a gravel road. On physical examination, muscle weakness was unremarkable, deep tendon reflexes were generally decreased, and an abnormal sensation was noted in the distal extremities. Blood test results revealed M-proteinemia of the IgM kappa-type, and CSF analysis revealed elevated protein levels (Table 3). The NCS revealed demyelination in multiple nerves, and waveforms were not evoked in the sensory nerves (Table 4, Figure 2).

**Table 3:** Results of the blood and CSF analysis in case two

Blood examination	Results	Normal range
White blood cells (/µL)	9,240	3300-8600
Hb (g/dL)	15.5	11.6-14.8
Platelets (×10^4^ /µL)	35.5	15.8-34.8
Creatinine (mg/dL)	0.53	0.46-0.79
HbA_1c_ (%)	7.4	4.3-5.8
C-reactive protein (mg/dL)	0.06	0.00-0.14
Soluble IL-2 receptor (U/mL)	184	157-474
Antinuclear antibody (fold)	<40	<40
Anti-double-stranded DNA IgG antibody (U/mL)	＜10	<12
Anti-Sjögren's syndrome-A antibody (U/mL)	≦1	<10
Anti-Sjögren's syndrome-B antibody (U/mL)	≦1	<10
Cytoplasmic anti-neutrophil cytoplasmic antibody (IU/mL)	≦1	<3.5
Perinuclear anti-neutrophil cytoplasmic antibody (IU/mL)	≦1	<3.5
Anti-acetylcholine receptor antibody (nmol/L)	23	<0.2
CSF analysis	Results	Normal range
Protein (mg/dL)	286	15-45
Glucose (mg/dL)	93	50-75
White blood cell count (/µL)	1	<5
Mononuclear cell (/µL)	1	
Polymorphonuclear cell (/µL)	0	
Albumin (mg/dL)	195	4.1-5.1
IgG (mg/dL)	19.4	
IgG index	0.562	<0.7

**Table 4:** Results of NCS in case two NCS: Nerve conduction studies

Nerve	Distal latency	Amplitude distal/proximal	Velocity	F-latency
Motor	ms	mV	m/s	ms
Median	8.5	3.0 / 2.7	37.3	37.7
Ulnar	5.8	5.4 / 4.8	50.0	30.2
Tibial	13.3	0.4 / 0.5	28.2	
Sensory	ms	μV	m/s	ms
Median	Not evoked	Not evoked	Not evoked	Not evoked
Ulnar	Not evoked	Not evoked	Not evoked	Not evoked
Sural	Not evoked	Not evoked	Not evoked	Not evoked

The patient's symptoms partially improved after the IVIG treatment. Myelin-associated glycoprotein (MAG) antibody-positive neuropathy was diagnosed based on the results of serum MAG antibody testing and nerve biopsies.

## Discussion

We encountered two cases of MG with M-proteinemia and demyelinating neuropathy. Both patients were diagnosed with early-onset MG in their 20s and developed M-proteinemia and demyelinating neuropathy in their 60s after thymectomy. Both patients received long-term immunosuppressive drug treatment.

Muscle weakness and fatigue are the typical symptoms of MG. These symptoms are variable and sometimes relapse [3]. Therefore, weakness of the extremities or fatigue in patients with MG is often considered a relapse of MG. The two cases reported in this study showed that lower limb symptoms and gait disturbances occurred in patients with MG due to neuropathy with M-proteinemia. If the motor symptoms in patients with MG worsen, the possibility of neuropathy should be considered.

To date, few cases of MG complicated with M-proteinemia and demyelinating neuropathy have been reported. In our cases, M-proteinemia was thought to be caused by DLBCL in case one and MAG antibody-positive neuropathy in case two. The M-proteinemia was triggered by immune cell dysfunction in both cases. A previous study reported an increased incidence of autoimmune diseases and malignancies after thymectomy [4]. Thymectomy limits T-cell production and increases the clonal expansion of antigen-presenting naïve T-cells in peripheral tissues. This process increases the possibility of self-sensitized T-cell proliferation and the risk of autoimmune disease onset [5]. Furthermore, an increased number of regulatory T-cells, as compensation for elevated self-antibodies [6], leads to the suppression of CD4-positive and CD8-positive cells, which increases the incidence of malignancies [7]. In the two cases reported here, thymectomy at a young age may have been associated with M-proteinemia and demyelinating neuropathy.

Long-term treatment with immunosuppressive drugs may also be a risk factor for malignancies and demyelinating neuropathy [8]. Calcineurin inhibitors such as TAC cause an increase in IL-6, TGF-β1, and vascular endothelial growth factor, which can be a risk factor for malignancies [9]. Hashimoto et al. also reported that advanced age and TAC use were risk factors for the development of lymphoma [10], consistent with case one reported here. As this study reports only two cases, we cannot confirm whether thymectomy and long-term treatment with immunosuppressive drugs are associated with M-proteinemia and demyelinating neuropathy. Therefore, further investigations are warranted.

## Conclusions

Here, we reported two cases of MG complicated by M-proteinemia and demyelinating neuropathy. Lower limb symptoms and gait disturbances in MG patients can easily be thought of as worsening their MG symptoms. However, the possibility of concomitant neuropathy should be considered when these symptoms worsen in patients with MG. Patients with early-onset MG who have received long-term treatment with immunosuppressive drugs after thymectomy may have a risk of developing late-onset demyelinating neuropathy with M-proteinemia.
